# Medication Therapy Management Guided by TIME Criteria in Nursing Home Residents: A Before–After Observational Study

**DOI:** 10.3390/jcm15093222

**Published:** 2026-04-23

**Authors:** Suna Avci, Aysegul Gundogan, Ayten Basak Karaakin Dinar, Ali Erol, Melike Yazici, Canberk Berkay Mert, Kubra Cingar Alpay, Gokalp Kurthan Avlagi, Meryem Merve Oren Celik, Ulev Deniz Erdincler, Gulistan Bahat

**Affiliations:** 1 Division of Geriatric Medicine, Department of Internal Medicine, Cerrahpasa Faculty of Medicine, Istanbul University-Cerrahpasa, Istanbul 34098, Türkiye; kubracingar@gmail.com (K.C.A.); gokalpkurthan@gmail.com (G.K.A.); denizsuna.erdincler@gmail.com (U.D.E.); 2 Department of Internal Medicine, Yalova Training and Research Hospital, Yalova 77100, Türkiye; gundoganaysegul@gmail.com; 3 Department of Internal Medicine, University of Health Sciences, Bursa Yuksek Ihtisas Training and Research Hospital, Bursa 16350, Türkiye; bkaraakin@gmail.com (A.B.K.D.); alierol625@gmail.com (A.E.); cbmert@gmail.com (C.B.M.); 4 Department of Internal Medicine, Kocaeli University School of Medicine, Izmit 41001, Türkiye; drmelikeyzc@gmail.com; 5 Department of Public Health, Istanbul Medical Faculty, Istanbul University, Istanbul 34093, Türkiye; meryem.oren@istanbul.edu.tr.oren; 6 Division of Geriatrics, Department of Internal Medicine, Istanbul Medical Faculty, Istanbul University, Istanbul 34093, Türkiye; gbahatozturk@yahoo.com

**Keywords:** potentially inappropriate medication use, TIME criteria, nursing home, outcome, falls

## Abstract

**Objectives:** Inappropriate medication use is highly prevalent in nursing home residents and contributes to adverse drug events, falls, and increased healthcare utilization. The Turkish Inappropriate Medication use in oldEr adults (TIME) criteria provide a comprehensive framework for identifying both potentially inappropriate medications and prescribing omissions. We aimed to evaluate the outcomes of a Medication Therapy Management (MTM) intervention by use of TIME criteria among nursing home residents in Türkiye. **Methods:** This single-arm before–after observational study included 232 adults aged 60–110 years residing in the Bursa Metropolitan Municipality Nursing Home. Medication use patterns were evaluated using the TIME criteria and prescriptions were optimized through a Medication Therapy Management (MTM) intervention. Fall frequency and healthcare utilization outcomes were recorded during the 1 year before and after MTM implementation. **Results:** Following implementation of the Medication Therapy Management (MTM) approach based on TIME criteria, the median number of medications increased from 5 (IQR: 3–8) to 8 (IQR: 5–10) over one year (*p* < 0.001). During the post-implementation period, fall frequency, emergency department visits, family medicine visits, and non-geriatric specialist visits were lower compared with the pre-intervention year, whereas hospitalization rates did not significantly change. **Conclusions:** Among nursing home residents, the total number of medications was higher and fall frequency and selected healthcare utilization measures were lower during the year following implementation of MTM based on TIME criteria. These findings suggest that MTM-guided prescription optimization may be associated with changes in clinical and healthcare utilization outcomes; however, the absence of a control group precludes causal inference, and randomized controlled trials are required to confirm these associations.

## 1. Introduction

Potentially inappropriate medication use (PIM) refers to the prescribing of medications in which potential harms outweigh expected benefits in older adults, whereas potential prescribing omission (PPO) denotes the failure to initiate clinically indicated treatments. These two domains constitute the core components of so-called “inappropriate prescribing” [1]. Inappropriate prescribing is common among older adults, particularly in institutional care settings, and leads to serious clinical consequences [2]. As a major global public health concern, inappropriate prescribing contributes substantially to adverse drug events, functional decline, falls, and unplanned hospital admissions [3,4]. Therefore, it is imperative that older adults, their families, and healthcare systems no longer bear the burden of inappropriate prescribing [5]. The rapid increase in the older population worldwide makes the quality and safety of medication prescribing in later life increasingly critical [6].

Prescription review is a structured process that includes verification of disease diagnoses, comprehensive evaluation of current medications, discontinuation of unnecessary or harmful drugs, and initiation of evidence-based treatments when clinically indicated but omitted [7]. Several explicit tools have been developed to support clinicians in identifying inappropriate prescribing, including the Beers Criteria, which focuses solely on PIM, and the STOPP (Screening Tool of Older Person’s Prescriptions) and START (Screening Tool to Alert to Right Treatment) criteria, which address PIM and PPO, respectively [8,9]. The Turkish Inappropriate Medication use in oldEr adults (TIME) criteria were developed for international use to identify inappropriate prescribing in older individuals, taking into account commonly prescribed medications, brand availability, and health system characteristics. Unlike other tools, the TIME criteria provide a comprehensive framework by simultaneously addressing both overprescribing and underprescribing. The TIME criteria consist of two complementary components: TIME-to-STOP, which identifies potentially inappropriate medications, and TIME-to-START, which highlights clinically indicated but omitted treatments [10].

Optimizing prescriptions for older adults is essential for improving the quality of care and preventing adverse drug effects [11]. Exposure to both PIM and PPO has been associated with increased mortality, falls, and healthcare utilization, highlighting the need for regular and comprehensive evaluation of pharmacotherapy in older populations [2,12,13]. Discontinuing inappropriate prescriptions in older adults is feasible, well tolerated and provides clinically meaningful benefits [14]. The clinical outcomes of interventions targeting inappropriate prescription use are frequently assessed in terms of changes in falls, emergency department visits, primary care visits, geriatric and other specialist clinical visits due to PIM and PPO relationships [3,14,15,16,17]. However, prospective evidence demonstrating the long-term outcomes of reducing inappropriate prescribing remains limited and there is a notable lack of systematic guidance to support clinicians in effectively implementing prescribing processes [18]. There is also a significant research gap regarding the long-term clinical effects of interventions targeting inappropriate medication use based on TIME criteria, an important tool that can support clinicians in prescription optimization. Therefore, there is an increasing need for effective, evidence-based interventions to optimize medication prescribing practices in nursing home settings.

The aim of this study was to evaluate the effects of MTM intervention, guided by the TIME criteria, on fall frequency and health care utilization among nursing home residents in Türkiye.

## 2. Materials and Methods

This was a single-arm before–after observational study conducted at the Bursa Metropolitan Municipality Nursing Home between March 2019 and January 2022, a period that encompassed the early phase of the COVID-19 pandemic. A total of 232 nursing home residents aged 60 years and older who underwent detailed geriatric assessments and received MTM intervention were included in the study. Given that the study period overlapped with the COVID-19 pandemic, healthcare utilization patterns may have been influenced independently of the intervention.

Ethical approval for the study was obtained from the Ethics Committee of Bursa Specialized Training and Research Hospital (Approval No: 2011-KAEK-25/2022-01/17, Date: 26 January 2022). The clinical activities described in this study were initially carried out as part of routine geriatric care and a structured Medication Therapy Management (MTM) service in the nursing home setting and were not originally designed as a formal research study. All participants (or their legal representatives) provided written and verbal informed consent prior to inclusion, and patient confidentiality and data protection were strictly maintained throughout the process. Following the completion of the clinical follow-up period, the data were organized for scientific evaluation, and ethical approval was subsequently obtained prior to data analysis and manuscript preparation. The study was conducted in accordance with the principles of Good Clinical Practice and the Declaration of Helsinki. Written and verbal informed consent was obtained from all participants who agreed to participate in this study.

Based on the descriptive statistics reported by Fog et al. [19], a priori power analysis was conducted using G*Power 3.1.9.7 [20]. The analysis indicated that a minimum of 159 participants was required to achieve 90% statistical power at a significance level of 0.05 (two-tailed). The criteria for inclusion in the study group were defined as: agreeing to participate in the study, having at least one chronic disease requiring regular medication, having completed follow-up assessments and being aged 60 years or older. Since the minimum age for admission to nursing homes in Türkiye is set at 60 years and older, the age threshold for this study was defined as 60 years [21]. Exclusion criteria were refusal to participate in the study, death during the study period, permanent discharge from the institution, incomplete follow-up data and receipt of palliative care due to terminal illness.

A total of 256 residents were initially assessed for eligibility. Of these, 24 were excluded for the following reasons: refusal to participate (*n* = 1), death during the study period (*n* = 2), permanent discharge from the institution (*n* = 11), incomplete follow-up data (*n* = 8), and transition to palliative care due to terminal illness (*n* = 3). The remaining 232 residents who completed both baseline and post-intervention assessments constituted the final study cohort. It should be noted that the exclusion of residents who died or left the facility before completing follow-up may have resulted in a relatively healthier analytic sample, which could lead to an overestimation of the intervention’s benefits. Participants were selected through consecutive sampling of all residents who met the inclusion criteria and were living in the facility during the baseline assessment period (September 2019–February 2020). The median length of stay of 6.5 years (IQR: 4–12) reflects the demographic composition of the study population at the time of enrolment and was not used as a selection criterion. Although the interquartile range begins at four years, 30 residents had been admitted within the preceding one to three years. The predominance of long-term residents is consistent with the low turnover rate typically observed in publicly funded nursing homes in Türkiye.

Nursing home visits, which began in March 2019, were implemented on a weekly basis for eight hours. During the time spent at the facility, one-on-one interviews were conducted with each resident to form the basic assessments required for the first phase of the study. Each assessment with a resident lasted approximately 30 min and an average of six residents were interviewed per week. The initial phase assessments for all residents were completed in February 2020. As the start of the study coincided with the COVID-19 pandemic, visits to nursing home residents also changed during the pandemic period and weekly visits were discontinued in March 2020 due to COVID-19 pandemic restrictions following the initial phase assessments. During this period, follow-ups were mostly conducted via remote telephone calls, short monthly visits of two hours and, when necessary, clinical appointments for residents. The follow-up period was completed for each resident and the final assessments were completed in February 2021, generating the study data. The study was conducted in three stages: initial assessment, intervention with MTM and post-intervention assessments.

### 2.1. Baseline Assessment

Baseline assessments were conducted during routine geriatric assessment visits which were conducted weekly between September 2019 and February 2020. We recorded sociodemographic characteristics, comorbidities, functional status, length of stay in the nursing home and medication use. Data regarding the presence of comorbidities were obtained from the medical records of each resident at the nursing home and from the e-Nabız system (the Turkish Ministry of Health’s personal health record platform). Current diagnoses, clinical findings and medication information were reviewed and recorded together. New disease diagnoses identified during the geriatric assessment were determined and necessary treatments were arranged. In the baseline assessment, data on retrospective healthcare utilization were recorded by reviewing both institutional nursing home records and national electronic health system records.

It should be noted that healthcare utilization data for the pre-intervention period were ascertained retrospectively from existing records, whereas post-intervention data were collected prospectively during the follow-up period. To minimize potential data-collection asymmetry, the same data sources—institutional nursing home records and the national e-Nabız electronic health record system—were used for both periods. These systems maintain standardized, routinely updated logs of emergency department visits, outpatient consultations, and hospitalizations, thereby reducing the likelihood of differential ascertainment bias between the two assessment periods.

The variables used to assess residents’ clinical status were history of falls, emergency department visits, family doctor visits, specialist visits for other branches, and hospital admissions. Falls were defined as any unintentional descent to the ground, floor, or a lower level, regardless of the presence of injury or need for medical attention, including slips, trips, and falls from standing, sitting, or bed, as recorded in institutional nursing home records. The proportion of residents who had at least one visit for each type of service and the total number of visits were recorded.

Frailty was assessed using a modified version of the Fried frailty phenotype, in which selected components were adapted to the clinical setting [22]. Unintentional weight loss was defined as a loss of ≥4.5 kg or ≥5% of body weight within the preceding year. Exhaustion was assessed based on self-reported persistent fatigue, defined as frequent feelings of exhaustion or low energy during daily activities. Physical activity level was evaluated using a simplified self-reported assessment of habitual physical activity, rather than the Minnesota Leisure Time Physical Activity Questionnaire. Muscle strength was measured by handgrip strength using a dynamometer (TAKEI GRIP D Model [T.K.K. 5401]; Takei Scientific Instruments Co., Ltd., Tokyo, Japan) and interpreted using fixed sex-specific cut-off values (<30 kg in men and <20 kg in women) for the frailty phenotype. Gait speed was assessed over a standardized 4 m walking distance at usual pace and classified according to sex- and height-specific thresholds. Participants meeting 3–5 criteria were classified as frail, those meeting 1–2 criteria as pre-frail, and those meeting none as robust.

Nutritional status was assessed according to the Global Leadership Initiative on Malnutrition (GLIM) criteria. Reduced muscle mass was assessed using bioelectrical impedance analysis (BIA) performed with a Tanita TBF-300 (Tanita Corporation, Tokyo, Japan) device, skeletal muscle mass index was calculated, and sex-specific cut-off values recommended by the GLIM consensus were adopted. Based on the presence of phenotypic parameters (weight loss, low body mass index (BMI) or reduced muscle mass) and etiologic parameters (reduced nutrient intake/assimilation or inflammation), participants were categorized as having “normal nutrition”, “stage 1 malnutrition,” or “stage 2 malnutrition” [23].

Sarcopenia was assessed according to the EWGSOP2 diagnostic algorithm. The same handgrip strength measurements were used and interpreted according to the EWGSOP2 gender-specific cut-off values (<27 kg for men and <16 kg for women). Muscle mass was assessed using BIA with the same device applied across all assessments, and low muscle mass was defined according to the cut-off values recommended by EWGSOP2. Physical performance was assessed using usual gait speed with a cut-off value of ≤0.8 m/s. Sarcopenia was classified into four groups: ‘no sarcopenia’, ‘possible sarcopenia’, “sarcopenia” and ‘severe sarcopenia’ [24].

Functional mobility status was categorized as fully independent, ambulatory with support, wheelchair-dependent or bedridden [25]. Weight was measured using a calibrated digital scale in ambulatory residents and a wheelchair/chair scale in non-ambulatory residents. Standing height was measured with a stadiometer in residents who were able to stand upright. In residents unable to stand or maintain an upright posture, stature was estimated using knee height measurements and standard equations. BMI was calculated by dividing body weight in kilograms by the square of height in meters (BMI = weight (kg)/height (m)^2^).

### 2.2. Medication Therapy Management Intervention

MTM was conducted by a specialist geriatrician (S.A.) who provided regular geriatric consultation services at the nursing home. Each resident’s entire medication regimen was comprehensively reviewed during routine geriatric clinic visits, and the clinical indications and medical diagnoses for each medication were evaluated. All active pharmaceutical ingredients, including those in combination therapies used by each individual, were examined separately and the total number of medications was recorded. Only medications with systemic effects were evaluated; topical, ophthalmic, nasal or rectal medications were not included in the total number of medications. Each medication was then systematically reviewed according to the TIME criteria to assess the appropriateness of its use [10,26]. Both PIMs (violations of TIME-to-STOP) and PPOs (violations of TIME-to-START) were identified and recorded. Drug–drug interactions, drug–disease interactions, and renal and hepatic function status were also thoroughly evaluated.

This process was conducted according to a structured protocol based on the TIME criteria and current clinical guidelines, and all medication changes were documented systematically. Based on a comprehensive review, we developed individualized medication management plans, taking into account the patient’s current health status and clinical stability, life expectancy and treatment goals, previous adverse drug reactions, patient and carer preferences, and a risk–benefit assessment for each medication change. Medication modifications were implemented gradually over a period of 6–12 months. High-risk medications (benzodiazepines, antipsychotics, anticholinergics, etc.) were tapered off gradually to minimize withdrawal effects. Similarly, medications without a clear indication were discontinued once clinical stability was achieved. Where appropriate, alternative non-pharmacological interventions were recommended. Indicated medications were administered as evidence-based preventive medications (calcium/vitamin D for osteoporosis, antiplatelet or anticoagulant therapy for cardiovascular disease, etc.), and treatment adjustments were made according to current clinical guidelines. During routine visits and telephone follow-up, residents’ side effects and clinical responses were monitored. Medication adherence was verified through nursing staff reports and medication administration records. Dose adjustments were made based on clinical response and tolerability.

### 2.3. Post-Intervention Assessments

Follow-up evaluations were conducted for all participants, ensuring that at least one year had passed since the start of MTM treatment. During this post-intervention one-year period, the number of documented falls and the patterns of healthcare utilization were evaluated and recorded.

### 2.4. Statistical Analysis

All analyses were performed using IBM SPSS Statistics version 27.0 (IBM Corp., Armonk, NY, USA). A *p*-value < 0.05 was considered statistically significant. The normality of continuous variables was assessed using histograms and Q–Q plots. Descriptive statistics were presented as mean ± standard deviation for normally distributed continuous variables, median (25th–75th percentile) for non-normally distributed continuous variables and frequency (percentage) for categorical variables. For repeated measures, paired t-tests were used for normally distributed continuous variables, while the Wilcoxon signed-rank test was applied for those not normally distributed. The McNemar test was used to compare paired categorical variables.

## 3. Results

The mean age of nursing home residents was 75.0 ± 8.3 years and 72.8% (*n* = 169) were male. The median length of stay in the nursing home was 6.5 (4–12) years. The median number of comorbidities per individual was 6 (IQR: 4–8). The most frequently identified comorbidities were hypertension (67.2%), coronary artery disease (51.3%), urinary incontinence (42.2%), osteoporosis (41.4%) and chronic renal failure (36.6%) (Table 1).

Mobility status was as follows: 25.0% walked with support, 9.1% used a wheelchair, and 12.1% were bedridden. According to the Fried Frailty Scale, 50.9% of the individuals were classified as frail and 36.2% as pre-frail. Based on the GLIM criteria, 15.8% of the participants had stage 1 malnutrition and 7.2% had stage 2 malnutrition. In the sarcopenia assessment, severe sarcopenia was identified in 25.2% of the residents (Table 1).

Application of the TIME-to-START criteria identified clinically indicated but previously omitted therapies in a substantial proportion of residents. Overall, 82.3% of participants had at least one new medication initiated during the MTM process. The most frequently initiated treatments were vitamin D and calcium supplementation (44.4%), antiresorptive or anabolic agents for osteoporosis (23.2%), oral nutritional supplements for malnutrition (22.7%), and antihypertensive therapy (22.8%). Cardiovascular preventive therapies included antiplatelet agents (12.0%), statins (5.17%), and oral anticoagulant therapy (2.6%) in residents with chronic non-valvular atrial fibrillation based on CHA_2_DS_2_-VASc assessment. ACE inhibitor therapy was initiated in 1.29% of residents with systolic heart failure (ejection fraction ≤ 40%) or ST-elevation myocardial infarction. Neuropsychiatric and cognitive therapies included antidepressants (9.9%), cholinesterase inhibitors in early-to-moderate Alzheimer’s disease (5.6%), and memantine in moderate-to-severe disease (6.5%). Respiratory treatments were introduced in 3.45% of residents with asthma or chronic obstructive pulmonary disease, including inhaled bronchodilators and inhaled corticosteroids when clinically indicated. Vaccination recommendations incorporated within the TIME-to-START framework were implemented in 93.5% of residents.

According to the TIME-to-STOP criteria, 56.4% of residents had at least one medication discontinued as part of the MTM intervention. The most frequently discontinued drug classes were medications with a high anticholinergic burden (12.0%), long-term non-steroidal anti-inflammatory drugs (8.1%), nootropic or cognitive enhancers (6.9%), intensive glucose-lowering therapies in frail patients (6.4%), thiazide diuretics (5.6%), and proton pump inhibitors prescribed without a clear indication (5.1%). In addition, psychotropic medications associated with an increased risk of falls were discontinued in 4.3% of residents. Other commonly discontinued treatments included selective serotonin reuptake inhibitors (3.8%), herbal supplements associated with bleeding risk (3.8%), long-term antivertigo agents (3.4%), non-steroidal anti-inflammatory drugs in patients with cardiovascular disease (3.0%), and long-acting sulfonylureas (3.0%).

Following the MTM intervention, the median number of medications used by participants increased from 5 (IQR: 3–8) to 8 (IQR: 5–10) over the one-year period (*p* < 0.001), reflecting the combined effect of initiating indicated treatments according to the TIME-to-START criteria and discontinuing inappropriate medications based on the TIME-to-STOP criteria. To further contextualize this finding, the net medication change reflected two concurrent processes: new medications were initiated in 82.3% of residents according to TIME-to-START criteria, while at least one medication was discontinued in 56.4% according to TIME-to-STOP criteria. Because TIME-to-START-driven initiations were more prevalent than TIME-to-STOP-driven discontinuations, the net effect was an increase in the total medication count.

The proportion of residents with a history of falls decreased from 23.7% to 15.9% (*p* = 0.026). Similarly, the number of emergency department visits declined significantly from 1 (IQR: 0–3) to 0.5 (IQR: 0–2) (*p* < 0.001). Visits to family medicine services also decreased, with the proportion falling from 36.2% to 25.0% (*p* = 0.001) and the number of family medicine visits decreasing from 0 (IQR: 0–2) to 0 (IQR: 0–0.5) (*p* < 0.001). Visits to non-geriatric specialist clinics decreased both in frequency (from 86.6% to 74.6%, *p* < 0.001) and in median visit count (from 10 (IQR: 3.5–18) to 2 (IQR: 0–6), *p* < 0.001). Rates of hospitalization (*p* = 0.824) and the number of inpatient stays (*p* = 0.311) did not significantly change following the intervention. (Table 2). Regular geriatric consultations were delivered throughout the MTM process, supplemented by remote follow-up during pandemic restrictions.

## 4. Discussion

Medication reviews play a critical role in managing adverse drug reactions and enhancing medication safety among older adults [17]. In this study, we examined changes in fall frequency and healthcare utilization following MTM guided by TIME criteria in older adults living in a nursing home and observed favorable changes in both fall rates and healthcare utilization over a one-year period.

In older individuals with multiple morbidities, an increase in PPOs may lead to an increase in the number of medications as a result of addressing treatment gaps. Doherty et al. reported that slightly more than one-third of the study group had PPOs and that increasing age and number of chronic conditions were associated with START PPOs [3]. In this study, the increase in the median number of medications from 5 to 8 following the MTM intervention based on TIME criteria may appear paradoxical at first glance; however, it is consistent with the results of similar interventions reported in the literature, which showed an increase in the average number of medications prescribed following medication reviews [27,28]. A large-scale study involving 591,726 individuals reported that the number of prescriptions tended to increase following drug reviews [27]. Similarly, in older adults, an increase in the number of prescriptions not associated with clinically adverse effects and an increase in medication costs have been reported following medication review interventions [28]. The increase observed following medication reviews, particularly in high-prevalence conditions such as osteoporosis, hypertension and coronary artery disease, where treatment gaps were identified, suggests that evidence-based treatment gaps have been closed and PPOs corrected, with the initiation of calcium/vitamin D, antiplatelet or anticoagulant treatments [3,4]. The results of the present study, consistent with these findings, indicate that an increase in the number of medications does not always indicate inappropriate prescribing; rather, it can be considered a rational increase linked to the completion of incomplete treatments in diseases such as osteoporosis, hypertension, and malnutrition. These findings suggest that short-term and non-integrated medication review interventions may have limited effectiveness in modifying established prescribing patterns and that sustained, multidisciplinary approaches may be required to achieve meaningful improvements in prescribing appropriateness. Furthermore, it is known that the deprescribing process for medications such as benzodiazepines and antipsychotics exhibits a dynamic structure, with discontinuation progressing gradually, whereas the initiation of preventive treatments occurs more rapidly [29]. This asymmetry—in which treatment initiation outpaced deprescribing—reflects a well-recognized clinical reality in populations with a high burden of untreated conditions. However, the total number of medications alone does not tell the full story. A resident who was started on vitamin D and calcium for untreated osteoporosis, or on aspirin for known coronary artery disease, is not in the same situation as one whose medication list grew due to prescribing cascades or unnecessary duplications. This is an important nuance that raw medication counts fail to capture, and we believe that future work in this area would benefit from using measures such as the Medication Appropriateness Index to better differentiate between appropriate and inappropriate polypharmacy. Therefore, in the present study, it can be said that adding medications (vitamin D/calcium (44.4%), antiresorptive or anabolic agents (23.28%), antihypertensives (22.84%), etc.) to the prescription, which was initiated in 82.33% of residents simultaneously with the discontinuation of harmful medications, may have improved the clinical appropriateness of the medication regimen and contributed to better patient outcomes, even though the total number of medications increased. In parallel, the preferential discontinuation of high anticholinergic-burden drugs, fall-risk-increasing psychotropics, long-term NSAIDs and proton pump inhibitors without clear indications represents a clinically meaningful shift toward safer prescribing patterns that may plausibly underlie the observed reductions in falls and emergency department visits.

In this before–after study, we demonstrated that a TIME-based MTM intervention was associated with a significant reduction in falls and emergency department visits, together with a restructuring of healthcare utilization patterns in nursing home residents. However, it should be acknowledged that part of the observed changes in healthcare utilization may also have been influenced by pandemic-related changes in care-seeking behavior during the study period.

It has been reported that certain groups of drugs increase the risk of falls, which are one of the most significant causes of injury in individuals aged 65 and over. As medication use is a modifiable risk factor, regular medication reviews should be an integral part of fall prevention strategies for older adults [30]. While certain medications, such as daily vitamin D supplements, should be added to the prescription to reduce falls, medications that increase the risk of falling, such as antipsychotic-sedative drugs, should be appropriately removed from the prescription [16,31]. However, the evidence base regarding medication use and falls is heterogeneous and requires careful interpretation. In the literature, there are studies reporting a decrease in fall rates with drug interventions [14,16,17] or finding no association between drug intervention and falls [32,33,34], as well as a small number of studies reporting an increase in falls with drug intervention [5]. In a randomized controlled trial involving older adults who were highly frail and taking five or more medications, no significant difference in fall rates was observed between groups over a three-month period following the discontinuation of prescriptions guided by the STOPPFrail guidelines [33]. In another MTM intervention study, although the number of medications increasing the risk of falls in older adults was significantly reduced by the intervention, no significant decrease in the risk and rate of falls was observed [34]. In a systematic review conducted by Lee and colleagues, it was reported that discontinuing medications that increase the risk of falls over a 6–12-month period did not result in a significant change in the incidence of falls, the number of people who fell or the rate of fall-related injuries [32]. In an intervention study conducted with older adults in five nursing homes in Canada, although the rate of prescription discontinuation increased with electronic decision support for deprescribing, a simultaneous increase in falls was also observed. Therefore, it is recommended that prescription reduction be carried out gradually and with a focus on safety [5]. It has been reported that discontinuing sedatives and psychotic drugs using a specific algorithm among vulnerable nursing home residents has a positive effect on the rate of falls [14]. A study evaluating the discontinuation of anticholinergic and sedative drugs in older adults in nursing homes reported a significant reduction in the number of falls over a six-month period [16]. In this study, the reduction in the fall rate from 23.7% to 15.9% over a one-year period following MTM intervention based on TIME criteria (*p* = 0.026) suggests that medication optimization may contribute to a reduction in fall frequency, though this association needs to be confirmed in controlled studies. The reduction in falls observed in the current study appears to be consistent with the potential combined effect of discontinuing high-risk medications with TIME-to-STOP and adding appropriate treatments such as vitamin D and calcium for conditions like osteoporosis and malnutrition with TIME-to-START; however, longer follow-up is considered necessary to confirm the continuity and causality.

Prospective data based on STOPP and START criteria have shown that exposure to any STOPP PIMs and exposure to two or more START PPOs are associated with an increase in emergency department visits [15]. Similarly, a large study involving 3619 older adults reported that patients with inadequate treatment according to START criteria made more emergency department visits over time [3]. These data suggest that addressing inadequate treatments and reducing inappropriate medications may have a positive effect on emergency department visits. However, the literature on the effect of medication adjustment interventions on emergency department visits is not homogeneous. In a study conducted among high-risk older individuals, it was reported that although the frequency of inappropriate prescriptions was significantly reduced, no difference was observed in emergency visits at 7 and 30 days [35]. Similarly, in an intervention involving older patients on polypharmacy, medication review was reported not to alter emergency department visit rates during a 4-month follow-up period [36]. Other results also report that medication reviews do not change emergency department revisit rates [37,38], but a study conducted among older adults attending the emergency department after a fall showed that medication review interventions were successful in reducing emergency visits for all causes [39]. In a study based on STOPP/START criteria across different centers, no significant difference was found in emergency visits and hospitalizations across the study group as a whole. However, it was reported that a decrease in emergency visits was observed in centers where treatment recommendations were accepted at a high rate [40]. This heterogeneous picture suggests that the effect of interventions depends not only on the criteria used but also on the acceptance rate of recommendations, the intensity of the intervention, the follow-up period and the organization of care. The reduction in emergency department visits observed in the current study is consistent with the hypothesis that the systematic and comprehensive structure of MTM based on TIME criteria may contribute to lower acute healthcare utilization, although this finding must be interpreted cautiously, given the concurrent pandemic and the absence of a control group. Reducing PIMs exposure with the TIME-to-STOP component, completing PPOs treatments with TIME-to-START and increasing planned follow-up after the intervention may have reduced emergency visits by preventing some acute decompensations and drug-related adverse events, resulting in a more stable clinical course in the short term. On the other hand, non-pharmacological adjustments implemented as part of the geriatrician’s assessment may also have influenced the residents’ clinical status (e.g., nutritional and hydration optimization, exercise and mobility support, fall-prevention measures and environmental modifications). Apart from these factors, it should be noted that emergency department visits had already decreased during the period in which the study was conducted due to the COVID-19 pandemic [41].

The COVID-19 pandemic resulted in widespread reductions in emergency department attendance and outpatient visits across Türkiye and globally, driven by public health restrictions, patient avoidance behavior, and institutional access limitations [41]. As the post-intervention data collection period (approximately February 2020–February 2021) substantially overlapped with the pandemic, it is plausible that a proportion of the observed reductions in emergency department visits, family medicine consultations, and non-geriatric specialist visits reflects pandemic-related suppression of healthcare utilization rather than—or in addition to—the effect of the MTM intervention. This temporal overlap represents a significant confound that cannot be disentangled from the intervention effect within the current study design.

In contrast to emergency department visits, hospitalization rates did not change significantly after the intervention. Although a high proportion of residents received influenza and pneumococcal vaccinations, no significant change in all-cause hospitalization rates was observed, which may reflect the multifactorial nature of hospital admissions in frail nursing home populations and the limited ability of preventive interventions to modify this outcome within a one-year period. Inappropriate prescription use is a factor that determines not only drug quality but also the intensity and distribution of service use. It has been reported that inappropriate prescribing is associated with an increase in emergency and outpatient/other polyclinic visits [3,15]. MTM interventions are expected to increase referrals by directing patients to more planned and coordinated follow-up points (primary care, geriatric outpatient clinics, deprescribing/MTM clinics) [7,27,35], while reducing emergency department visits related to medication-related adverse events and scattered, recurring problem-focused specialist referrals [39,40] or at least prevent their increase [35,36,37,38]. This study observed a significant decrease in the overall number of outpatient visits following the MTM intervention based on TIME criteria, but also a remarkable restructuring in visit patterns. Specifically, the decrease in family medicine visits from 36.2% to 25.0% and the decrease in visits to other specialist outpatient clinics from 86.6% to 74.6% indicate a shift in the pattern of outpatient care utilization. Throughout the study period, residents received regular geriatric follow-up as part of the MTM process. These findings indicate that the follow-up burden was progressively redirected toward a more structured and coordinated model of care. Regular geriatric follow-up combined with MTM appeared to rationalize the medication regimen and was associated with a shift in the overall pattern of healthcare utilization. In the present study, the marked reduction in visits to family physicians and other non-geriatric outpatient clinics likely reflects a diminished need for fragmented, problem-driven consultations once patients were incorporated into a systematic geriatric follow-up framework. Beyond a simple reduction in visit numbers, MTM based on the TIME criteria was associated with a reorganization of care trajectories toward fewer unplanned referrals and a greater concentration of follow-up within geriatric services.

### Limitations

This study has a number of limitations that should be kept in mind when interpreting the findings. The most critical of these is the single-arm before–after design, which lacks a concurrent control group and therefore does not allow causal conclusions to be drawn. It is possible that the observed changes in falls and healthcare utilization are attributable to regression to the mean, secular trends, or confounders we were unable to measure, rather than to the intervention itself. Accordingly, all findings should be regarded as associations.

Because residents who died, were discharged, or moved to palliative care during follow-up were excluded from the analysis, the final cohort may over-represent healthier individuals, and this survivorship bias could have inflated the apparent benefits of the intervention. In addition, although the same institutional records and national e-Nabız system were used for both periods, baseline healthcare utilization was ascertained retrospectively while post-intervention data were collected prospectively, which may have resulted in differential ascertainment—particularly if earlier documentation was incomplete.

A further major confound is the overlap of the post-intervention period (approximately February 2020–February 2021) with the COVID-19 pandemic, which independently reduced emergency department attendance and outpatient visits across Türkiye. The observed reductions in healthcare utilization therefore cannot be attributed solely to the MTM intervention, and the true magnitude of any intervention effect may be smaller than estimated.

Several other methodological constraints should also be acknowledged. The change in PIM and PPO levels was not quantified, and the lack of a drug-class-level analysis limits interpretation of the increase in total medication count. Not all vaccines recommended by the TIME-to-START criteria could be administered, owing to supply limitations and patient-related barriers. The age threshold of 60 years, adopted to align with Turkish nursing home admission policy, may limit comparability with studies using the conventional cut-off of 65 years. The institutional setting with established geriatric services, together with the predominance of long-term residents, limits the generalizability of the findings to community-dwelling older adults, recently admitted residents, or healthcare systems organized differently. It should also be recognized that factors unrelated to the intervention—such as natural progression of disease, changes in nursing staff, and pandemic-driven institutional adjustments—may have independently shaped the outcomes observed during follow-up.

Notwithstanding these limitations, the present study adds to a limited evidence base on the clinical effects of TIME-based MTM in nursing home populations, and we hope it will serve as groundwork for future randomized, multicenter trials with extended follow-up periods.

## 5. Conclusions

This study suggests that medication management based on TIME criteria may be associated with favorable changes in fall frequency and healthcare utilization patterns in older adults living in nursing homes over a one-year period. The increase in the number of medications observed after the intervention possibly reflects the completion of incomplete treatments; the strengthening of geriatric-centered monitoring is consistent with a shift toward more coordinated care. Randomized controlled and community-based studies with a broader scope are needed to evaluate the long-term effects of MTM intervention and changes in prescribing on patient outcomes.

## Figures and Tables

**Table 1:** Sociodemographic, Clinical and Comorbidity Characteristics of Patients.

	*n* (%) (*n* = 232)
Age, years	75.03 ± 8.31
Sex	
Male	169 (72.84%)
Female	63 (27.16%)
Weight, kg	69.75 ± 15.18
Body mass index, kg/m^2^	24.85 (21.9–28.4)
Length of stay in nursing home, years	6.5 (4–12)
Number of comorbidities	5.94 ± 3.11
Comorbidities	
Hypertension	156 (67.24%)
Diabetes mellitus	70 (30.17%)
Chronic obstructive pulmonary disease	55 (23.71%)
Asthma	4 (1.72%)
Coronary artery disease	119 (51.29%)
Congestive heart failure	68 (29.31%)
Osteoarthritis	32 (13.79%)
Chronic renal failure	85 (36.6%)
Hyperlipidemia	49 (21.12%)
Malnutrition	58 (25.00%)
Polyneuropathy	31 (13.36%)
Dementia	46 (19.83%)
Depression	57 (24.57%)
Cerebrovascular disease	44 (18.97%)
Parkinson’s disease	16 (6.90%)
Sleep disorder	33 (14.22%)
Atrial fibrillation	15 (6.47%)
Peripheral artery disease	11 (4.74%)
Deep vein thrombosis	2 (0.86%)
Glaucoma	3 (1.29%)
Vertigo	6 (2.59%)
Epilepsy	8 (3.45%)
Hyperthyroidism	4 (1.72%)
Hypothyroidism	23 (9.91%)
Benign prostate hyperplasia	53 (22.84%)
Rheumatic diseases	9 (3.88%)
Gastritis/Peptic ulcer	16 (6.9%)
Chronic liver disease	3 (1.29%)
Osteoporosis	96 (41.4%)
Obstructive sleep apnea syndrome	3 (1.29%)
Urticaria	1 (0.43%)
Urinary incontinence	98 (42.2%)
Malignancy	17 (7.33%)
Walking capability	
Normal	125 (53.88%)
With support	58 (25.00%)
Wheelchair	21 (9.05%)
Bedridden	28 (12.07%)
Fried Frailty Scale score	3 (1–3)
Robust (0)	28 (12.84%)
Pre-frail (1–2)	79 (36.24%)
Frail (3–5)	111 (50.92%)
GLIM criteria	
No malnutrition	170 (76.92%)
Stage 1 malnutrition	35 (15.84%)
Stage 2 malnutrition	16 (7.24%)
Sarcopenia status	
No sarcopenia	53 (31.7%)
Probable sarcopenia	60 (35.9%)
Sarcopenia	12 (7.2%)
Severe sarcopenia	42 (25.2%)

**Table 2:** Summary of number of medications, body weight and hospital admission characteristics before and after medication therapy management.

	Before	After	*p*
Number of medications	5 (3–8)	8 (5–10)	**<0.001 ^‡^**
Body weight, kg	69.75 ± 15.18	69.77 ± 14.49	0.951 ^†^
Fall history	55 (23.71%)	37 (15.95%)	**0.026 ^§^**
Healthcare provider visit	204 (87.93%)	213 (91.81%)	0.137 ^§^
Total number of healthcare provider visits	16.5 (7–27)	8 (3–13)	**<0.001 ^‡^**
Emergency service visit	133 (57.33%)	116 (50.00%)	0.079 ^§^
Number of emergency department visits	1 (0–3)	0.5 (0–2)	**<0.001 ^‡^**
Family medicine visit	84 (36.21%)	58 (25.00%)	**0.001 ^§^**
Number of family medicine visits	0 (0–2)	0 (0–0.5)	**<0.001 ^‡^**
Non-geriatric specialist visit	201 (86.64%)	173 (74.57%)	**<0.001 ^§^**
Number of non-geriatric specialist visits	10 (3.5–18)	2 (0–6)	**<0.001 ^‡^**
Hospital stay (inpatient)	66 (28.45%)	69 (29.74%)	0.824 ^§^
Number of hospital stays (inpatient)	0 (0–1)	0 (0–1)	0.311 ^‡^

Descriptive statistics are presented using mean ± standard deviation for normally distributed continuous variables, median (25th percentile–75th percentile) for non-normally distributed continuous variables and frequency (percentage) for categorical variables. ^†^ Paired *t* test, ^‡^ Wilcoxon signed ranks test, ^§^ McNemar test. Statistically significant *p* values are shown in bold.

## Data Availability

The data that support the findings of this study are available from the corresponding author upon reasonable request.

## Abbreviations

MTM	Medication Therapy Management
TIME	Turkish Inappropriate Medication use in oldEr adults
PIM	Potentially inappropriate medication
PPO	potential prescribing omission
GLIM	Global Leadership Initiative on Malnutrition
BIA	bioelectrical impedance analysis
BMI	body mass index
